# Missing Value Imputation Method for Multiclass Matrix Data Based on Closed Itemset

**DOI:** 10.3390/e24020286

**Published:** 2022-02-16

**Authors:** Mayu Tada, Natsumi Suzuki, Yoshifumi Okada

**Affiliations:** 1 Division of Information and Electronic Engineering, Muroran Institute of Technology, 27-1, Mizumoto-cho, Muroran 050-8585, Hokkaido, Japan; 21043043@mmm.muroran-it.ac.jp (M.T.); 20043028@mmm.muroran-it.ac.jp (N.S.); 2 College of Information and Systems, Muroran Institute of Technology, 27-1, Mizumoto-cho, Muroran 050-8585, Hokkaido, Japan

**Keywords:** missing value imputation, multiclass matrix data, closed itemset, local feature space

## Abstract

Handling missing values in matrix data is an important step in data analysis. To date, many methods to estimate missing values based on data pattern similarity have been proposed. Most previously proposed methods perform missing value imputation based on data trends over the entire feature space. However, individual missing values are likely to show similarity to data patterns in local feature space. In addition, most existing methods focus on single class data, while multiclass analysis is frequently required in various fields. Missing value imputation for multiclass data must consider the characteristics of each class. In this paper, we propose two methods based on closed itemsets, CIimpute and ICIimpute, to achieve missing value imputation using local feature space for multiclass matrix data. CIimpute estimates missing values using closed itemsets extracted from each class. ICIimpute is an improved method of CIimpute in which an attribute reduction process is introduced. Experimental results demonstrate that attribute reduction considerably reduces computational time and improves imputation accuracy. Furthermore, it is shown that, compared to existing methods, ICIimpute provides superior imputation accuracy but requires more computational time.

## 1. Introduction

In data analysis, when there are missing values in the data, many analysis methods do not provide accurate results [1,2,3,4]. Therefore, handling missing values is a very important issue in data analysis [5,6,7].

There are two main approaches to handling missing values. First, if there is a small number of instances that contain missing values (e.g., samples or attributes in a matrix data), such instances can be deleted [2,8]. However, if there is a significant number of such instances, this approach should not be applied because it can result in the loss of important information. The second approach involves imputing the values of missing data based on their similarity to data patterns of other instances [9,10]. Data imputation facilitates the application of analytical methods to complete datasets without changing the size of the dataset. To date, many data imputation methods based on various algorithms, such as k-nearest neighbor [11,12], the least squares principle [13], random forest [14], decision tree [15], and naïve Bayes [16], have been proposed. Most previously proposed imputation methods use trends across all instances, i.e., the entire feature space, to estimate missing values. However, the feature space around each missing data item is likely to follow data patterns in local feature space. Therefore, it is important to estimate missing values based on local feature space.

Most existing approaches for dealing with missing values focus on single class datasets. Handling missing values in multiclass datasets requires techniques that utilize characteristic data patterns in each class.

The motivation of this study is to provide a high-accuracy method for missing value imputation using local feature space for multiclass matrix datasets. To this end, we propose an innovative approach based on closed itemset mining. This paper describes two data imputation methods, CIimpute and ICIimpute, based on closed itemsets that typically occur in each class. Note that we assume that rows, columns, and elements in matrix data correspond to samples, attributes, and data, respectively, and that each sample has a class label. A closed itemset is a subset of attribute values that commonly occur in a subset of samples in matrix data; thus, a closed itemset can be used to represent local features around a missing value. CIimpute estimates missing values using closed itemsets occurring in each class. However, closed itemset mining from matrix data is a combinatorial search problem for samples and attributes; therefore, the computational cost increases exponentially as the matrix data size increases. To address this problem, ICIimpute introduces an attribute reduction process to CIimpute. The proposed methods are evaluated using four UCI datasets [17] and compared with well-known existing methods.

The remainder of this paper is organized as follows. Section 2 describes closed itemsets. Section 3 explains the procedures of the proposed methods. Experiments conducted to evaluate the proposed methods are described in Section 4, and the experimental results and some observations are presented and discussed in Section 5. Conclusions and suggestions for future work are presented in Section 6.

## 2. Closed Itemset

A closed itemset is utilized to estimate missing values based on similarity of local feature space in matrix data. This section defines a closed itemset and describes the LCM algorithm applied to exhaustively enumerate closed itemsets.

### 2.1. Definition

Let I = {1, 2, …, n} be a set of items. A transaction database on I is defined as T = {s_1_, s_2_,…, s_m_} such that each s_i_ is included in I. Each s_i_ is called a transaction. A set P ⊆ I is called an itemset. A transaction including P is called an occurrence of P. The set of occurrences of P is expressed as T(P). The size of a set of occurrences for P is referred to as the frequency of P. An itemset P is called a closed itemset if no other itemset Q satisfies T(P) = T(Q), P ⊆ Q. For a given minimum support constant (hereafter θ), P is frequent if |T(P)| ≥ θ. A frequent and closed itemset is referred to as a frequent closed itemset.

**Figure 1:**
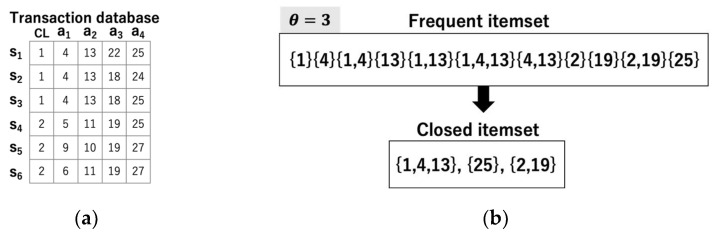
(**a**) Transaction database; (**b**) Frequent itemsets and closed itemsets extracted from (**a**).

In this study, a transaction database is represented by matrix data. In this matrix data, rows, columns, and elements are considered as transactions (hereafter samples), attributes, and items, respectively. Figure 1a shows a transaction database where each transaction has five items. Figure 1b shows the frequent itemsets and the closed itemsets when θ = 3. For example, {1, 4} and {4, 13} are frequent itemsets but are not closed itemsets because both itemsets are subsets of {1, 4, 13}, i.e., the maximal itemsets (i.e., closed itemsets) in the occurrence set {s_1_, s_2_, s_3_}.

### 2.2. LCM Algorithm

Mining closed itemsets using a naive full search requires considerable computation time because it involves a combinatorial search. In this study, we employ a fast and efficient LCM algorithm [18,19] that, depending on the size of the database, can enumerate frequent closed itemsets in linear time using a unique approach called prefix-preserving closure extension. This extension generates a new frequent closed itemset from the previously obtained itemset without duplication by the depth-first search technique; therefore, unnecessary non-closed frequent itemsets can be completely pruned.

## 3. Methods

In this section, we describe the proposed missing value imputation methods. Here, CIimpute estimates missing values using closed itemsets occurring in each class in multiclass matrix data, and ICIimpute introduces an attribute reduction process to CIimpute.

### 3.1. Preprocessing

**Figure 2:**
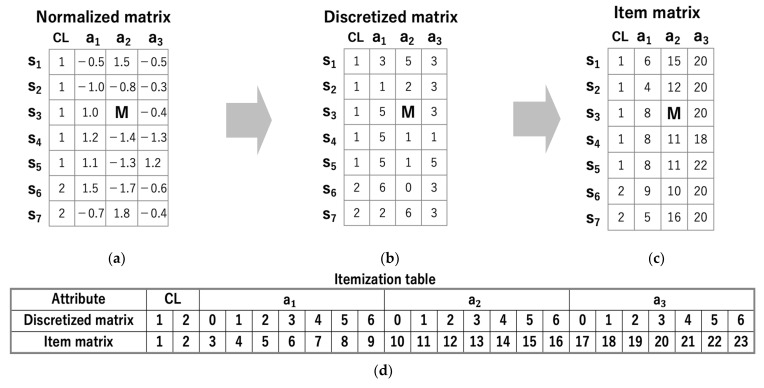
Preprocessing procedure. (**a**) Normalized matrix; (**b**) Discretized matrix transformed from (**a**); (**c**) Item matrix transformed from (**b**); (**d**) Itemization table.

Figure 2 shows the preprocessing procedure. The input data is a multiclass matrix with class labels, as shown in Figure 2a. Each row and column represent a sample and an attribute, respectively. Here, CL denotes the class label, and M represents a missing value. First, the elements of attributes other than CL are normalized in the column direction using z-score normalization. Next, the normalized matrix is transformed into a discretized matrix, as shown in Figure 2b. The element values of attributes other than CL are discretized evenly into k levels. In this study, k was set to 7 following the results reported in [20]. Finally, the discretized matrix is transformed into an item matrix, as shown in Figure 2c. The item matrix is constructed using the itemization table shown in Figure 2d. In the itemization table, each class and each discretized value correspond to a unique number, i.e., an item. Each class is assigned an item starting from 1, and each discretized value is assigned an item in order starting from the number of classes + 1. The item matrix can be constructed by the above procedure regardless of the number of divisions in discretization. We use the item matrix as input data (transaction data) to extract closed itemsets.

### 3.2. CIimpute: Closed Itemset-Based Imputation Method

**Figure 3:**
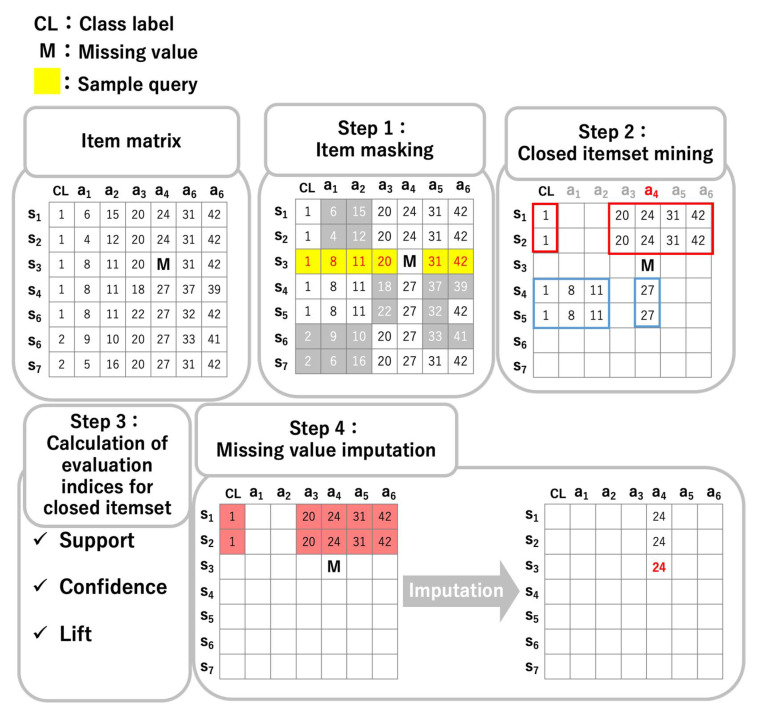
Procedure of CIimpute.

Figure 3 shows the procedure of CIimpute. CIimpute comprises four steps: (1) item masking, (2) closed itemset mining, (3) calculation of evaluation indices for the closed itemset, and (4) missing value imputation. The steps correspond to Step 1, 2, 3, and 4 in Figure 3, respectively.

#### 3.2.1. Step 1: Item Masking

A sample with missing values is referred to as a sample query. In Figure 3, sample s3 is a sample query. For each column, items between the sample query and the other samples are compared, and items that differ from the sample query are deleted because closed itemsets with such items cannot be used for missing value imputation. Item masking reduces computational time because closed itemsets with masked items do not need to be searched.

#### 3.2.2. Step 2: Closed Itemset Mining

After item masking, closed itemsets that include both the CL attribute and the attribute with a missing value are mined from the matrix data. The CL attribute is used to discriminate closed itemsets occurring in each class. In other words, the closed itemset including CL is a closed itemset occurring in the class CL. In contrast, the closed itemset without CL is a closed itemset occurring across multiple classes. The attribute including the missing value is utilized to estimate the missing value.

#### 3.2.3. Step 3: Calculation of Evaluation Indices for Closed Itemset

For the closed itemsets obtained in Step 2, the following three indices are calculated.

(1) $$\mathrm{support}\left(X\to Y\right)=\frac{\left|X\cup Y\right|}{\left|D\right|},$$

(2) $$\mathrm{confidence}\left(X\to Y\right)=\frac{\left|X\cup Y\right|}{\left|X\right|},$$

(3) $$\mathrm{lift}\left(X\to Y\right)=\frac{\mathrm{confidence}\left(X\to Y\right)}{\frac{\left|Y\right|}{\left|D\right|}},$$

where X is a set of items other than items of an attribute including the missing value, Y is the item of an attribute including the missing value, and D is the number of samples in the matrix data.

#### 3.2.4. Step 4: Missing Value Imputation

For the closed itemset with the maximum score in each index calculated in Step 3, the estimated value of the missing value, e(M), is computed as follows:

(4) $$e\left(M\right)=\mathrm{norm}\_\min\left(a_{M}\right)+\mathrm{clo}\_\mathrm{disc}\left(a_{M}\right)\times \mathrm{interval}\left(a_{M}\right),$$

where $a_{M}$ is an attribute including the missing value M, $\mathrm{norm}\_\min\left(a_{M}\right)$ is a function that returns the minimum value in the column of $a_{M}$ in the normalized matrix, $\mathrm{clo}\_\mathrm{disc}\left(a_{M}\right)$ is a function that returns the discretized value corresponding to the item in the column of $a_{M}$ of the closed itemset obtained in Step 3, and $\mathrm{interval}\left(a_{M}\right)$ is a function that returns the discrete interval of the column of $a_{M}$ in the normalized matrix.

### 3.3. ICIimpute: Improved Closed Itemset-Based Imputation Method

Closed itemset mining requires significant computation time due to the combinatorial problem. The LCM algorithm is a fast and efficient algorithm for a sparse transaction database (matrix data). In Section 3.2, sparse matrix data was generated by item masking in the column direction to improve the computational efficiency of closed itemset mining. However, the computational time required for closed itemset mining is also considerably influenced by the number of attributes.

**Figure 4:**
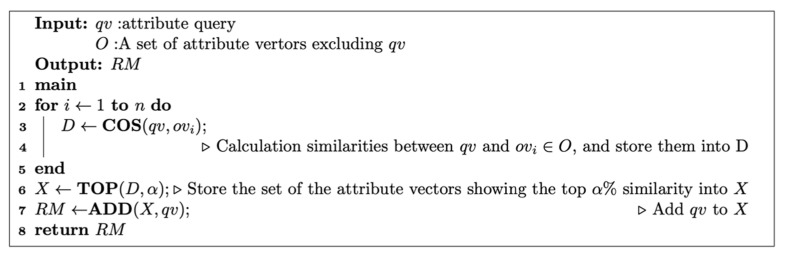
Pseudocode of the attribute reduction process.

Here, we describe ICIimpute, which introduces the attribute reduction process. The pseudocode of the attribute reduction process is provided in Figure 4. This process is part of the preprocessing described in Section 3.1. Input data for the attribute reduction process is a normalized matrix with CLs. The column vector of the attribute including the missing value is called an attribute query. First, a similarity measurement, i.e., cosine similarity, between the attribute query and the rest of the column vectors (hereafter attribute vectors) is performed. Subsequently, the attribute vectors showing the top α% similarity are extracted. The reduction rate is defined as (100 − α)%. Next, new matrix data is generated by adding these attribute vectors to the attribute query. Finally, the new matrix data is converted to an item matrix according to the procedure described in Section 3.1. Subsequent missing value imputation is performed according to the procedure described in Section 3.2. By executing the above process for all attribute queries, all missing values can be imputed. The goal of the attribute reduction process is to eliminate column vectors with low similarity to the attribute query. This process is expected to reduce the amount of computation because it reduces the search process for closed itemsets that do not contribute to missing value imputation.

## 4. Experiments

### 4.1. Dataset

Evaluation experiments were conducted using the UCI datasets listed in Table 1. Note, all four are multiclass matrix datasets.

### 4.2. Evaluation Method

Both CIimpute and ICIimpute require a minimum support constant θ as a parameter in the closed itemset mining. θ is generally set to a value of 2 or more. By setting a smaller θ, more computational time is required, but more closed itemsets available for missing value imputation can be obtained. The result of preliminary experiments under θ = 2 and 3 showed that θ = 3 provided almost the same number of closed itemsets in shorter computational time compared to θ = 2. Hence, in this study, θ was set to 3 in both CIimpute and ICIimpute.

The imputation accuracy and computation time of both proposed methods were evaluated experimentally by estimating randomly generated missing values. The imputation accuracy was evaluated using the root mean square error (RMSE) metric, which is calculated as follows:

(5) $$\mathrm{RMSE}=\sqrt{\frac{\sum_{i=0}^{n}{\left(x_{i}-x_{i}^{\prime }\right)}^{2}}{n}},$$

where n is the number of missing values, $x_{i}$ is an actual value, and $x_{i}^{\prime }$ is an estimated value. The imputation accuracy improves as the RMSE value approaches zero. The RMSE calculation and computational time measurement were performed on a workstation with an Intel(R) Core™ i7-9700 3.00 GHz processor with 8.00 GB RAM.

We conducted experiments to evaluate the proposed methods and to compare their performance to that of existing imputation methods, i.e., LSimpute [13], KNNimpute [11], and RF [14]. LSimpute is based on the least squares principle and utilizes correlations between both samples and attributes. KNNimpute imputes missing values using a weighted average of K other instances of a similar data pattern (nearest neighbors). RF is the random-forest-based imputation method.

## 5. Results and Discussion

### 5.1. Evaluation Results with Different Attribute Reduction Rates

For both CIimpute and ICIimpute, the RMSE values and computational times with different attribute reduction rates were evaluated. In this experiment, the average RMSE and average computational time for various reduction rates were calculated using 20 matrix data with 10% randomly generated missing values.

#### 5.1.1. Imputation Accuracy

**Figure 5:**
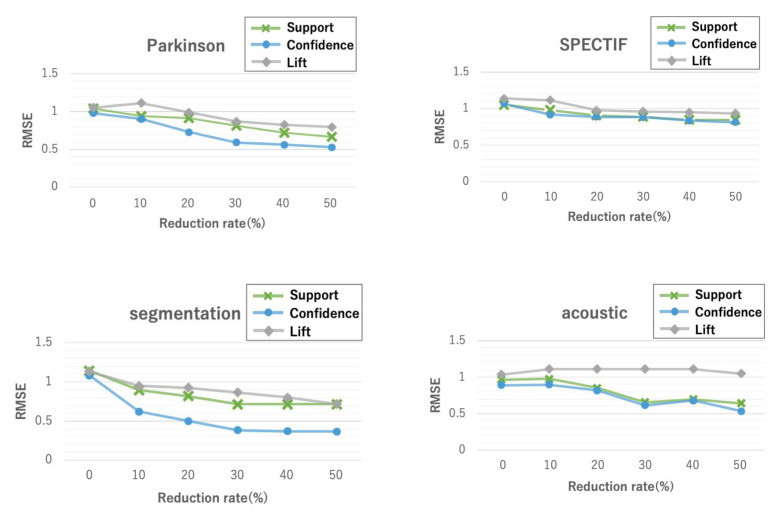
Imputation accuracy of the proposed methods on four datasets. A reduction rate of 0% indicates CIimpute; reduction rates of 10% or more indicate ICIimpute.

Figure 5 shows the RMSE for three evaluation indices, i.e., support, confidence, and lift, for different reduction rates on four datasets. In Figure 5, a reduction rate of 0% indicates CIimpute, and reduction rates of 10% or more indicate ICIimpute. Reduction rates greater than 50% were excluded from the results because, in some cases, no closed itemset that can be used for missing value imputation was extracted. As can be seen, the confidence score showed the best accuracy for all datasets. Accuracy tended to improve as the reduction rate increased. In particular, when comparing the RMSE of CIimpute (without attribute reduction) and ICIimpute (with attribute reduction) with reduction rates of 50%, statistically significant differences ($p<10^{-5}$) were observed in all data sets, which indicates that attribute reduction improves imputation accuracy.

#### 5.1.2. Computational Time

**Figure 6:**
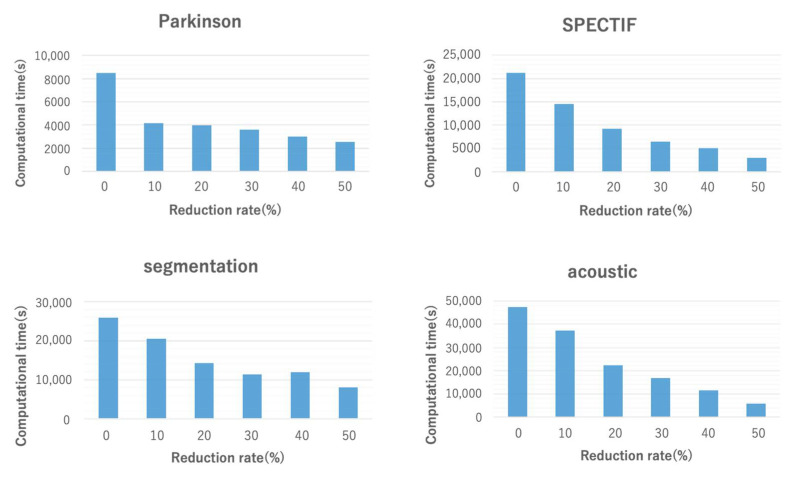
Computational time of the proposed methods. A reduction rate of 0% indicates CIimpute; reduction rates of 10% or more indicate ICIimpute.

Figure 6 shows the computational time for different reduction rates. For all datasets, the computational time tended to decrease as the reduction rate increased. In particular, compared to CIimpute, when the reduction rate was 50%, the computational time was dramatically reduced. As mentioned previously, the computational time incurred by closed itemset mining is strongly dependent on the number of attributes; as the number of attributes increases, the number of combinations of attributes to be checked increases rapidly. Attribute reduction significantly reduces the search space; consequently, the computational time is dramatically reduced.

### 5.2. Evaluation Results with Different Missing Value Rates

The RMSE and the computational time with different missing value rates were determined. Here, the reduction rate was fixed at 50%, and the evaluation index was the confidence level. In this experiment, the average RMSE and average computational time for different missing rates were calculated using 20 matrix data with randomly generated missing values.

#### 5.2.1. Imputation Accuracy

**Figure 7:**
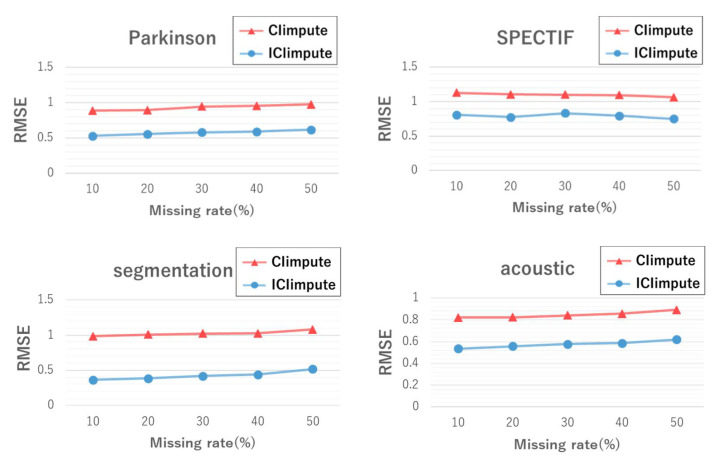
Imputation accuracy with different missing rates.

Figure 7 shows the RMSE values for CIimpute and ICIimpute with different missing rates. Missing rates greater than 50% were excluded from the results because no closed itemset that can be used for missing value imputation was extracted. ICIimpute showed statistically significant better imputation accuracy ($p<10^{-6}$) than CIimpute for all datasets. These results indicate that the attribute reduction process contributed to imputation accuracy regardless of the missing rate.

#### 5.2.2. Computational Time

**Figure 8:**
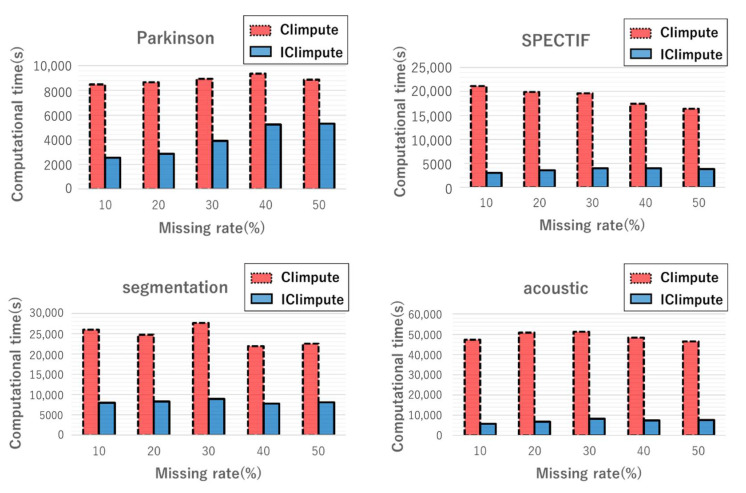
Computational time with different missing rates.

The computational times for CIimpute and ICIimpute with different missing rates are shown in Figure 8. This figure indicates that ICIimpute had shorter computational time than CIimpute regardless of missing rates. This is because the attribute reduction process drastically reduced the closed itemset mining search space. The results demonstrate that attribute reduction contributed to the reduction of computational time regardless of the missing rate.

### 5.3. Comparison with Existing Methods

The results presented in Section 5.1 and Section 5.2, demonstrate that, in terms of imputation accuracy and computational time, ICIimpute is superior to CIimpute. To further evaluate ICIimpute, we compared the average RMSE and average computational time of ICIimpute to KNNimpute, LSimpute, and RF. In ICIimpute, the reduction rate was fixed at 50%, and the evaluation index was the confidence level.

#### 5.3.1. Comparison of Imputation Accuracy

**Figure 9:**
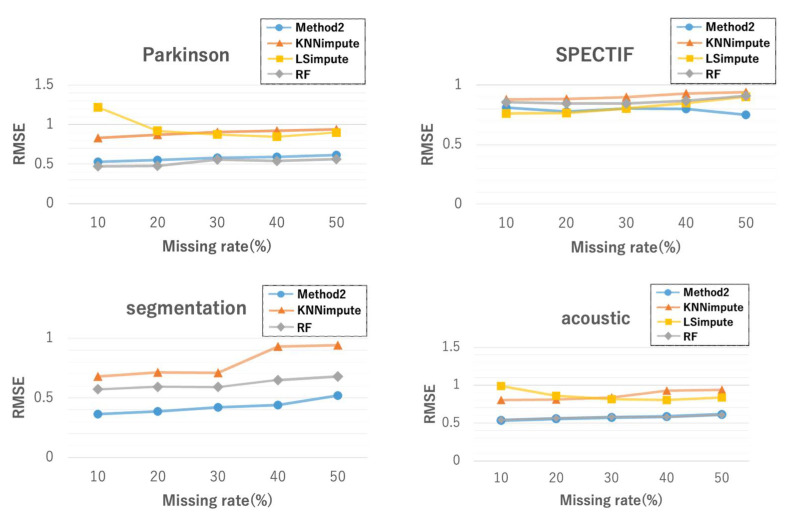
Comparison of imputation accuracy between ICIimpute and three existing methods.

Figure 9 shows the RMSEs when missing rates vary from 10% to 50%. The results for LSimpute with the segmentation dataset are not shown because the program terminated before completion. Overall, ICIimpute showed better imputation accuracy regardless of the missing rates compared to the other three methods. In particular, with the segmentation dataset, a statistically significant difference ($p<10^{-5}$) in the imputation accuracy was observed between ICIimpute and the other two methods. Furthermore, ICIimpute exhibited robust accuracy to the variations in the missing rates compared to the other methods.

#### 5.3.2. Comparison of Computational Time

Table 2 shows the computational times for ICIimpute, KNNimpute, LSimpute, and RF when the missing rate was fixed at 30%. As mentioned previously, the results for LSimpute with the segmentation dataset are not available because the program terminated before completion. For all datasets, ICIimpute required more computational time because it employs closed itemset mining, which includes a combinatorial search of attributes and samples in matrix data. Although data are not provided, similar results were observed with other missing rates.

### 5.4. Discussion

In high-dimensional spaces, it is difficult to obtain reasonable results because the distance between individual instances (samples or attributes) tends to be large due to the curse of dimensionality. In terms of missing value imputation, using the entire feature space in large matrix data may not always yield adequate estimates. However, our proposed closed itemset-based methods use local feature space, i.e., only the attribute set associated with the attribute containing the missing value. Therefore, the influence of most other attributes that are likely to become noise can be eliminated. CIimpute required significant computational time for a large-scale matrix. Thus, an attribute reduction process was introduced in ICIimpute. This process reduces the search space of the closed itemsets and focuses only on attributes that show similar data patterns to the attributes containing the missing values. Consequently, ICIimpute showed improved imputation accuracy and reduced computational time.

**Figure 10:**
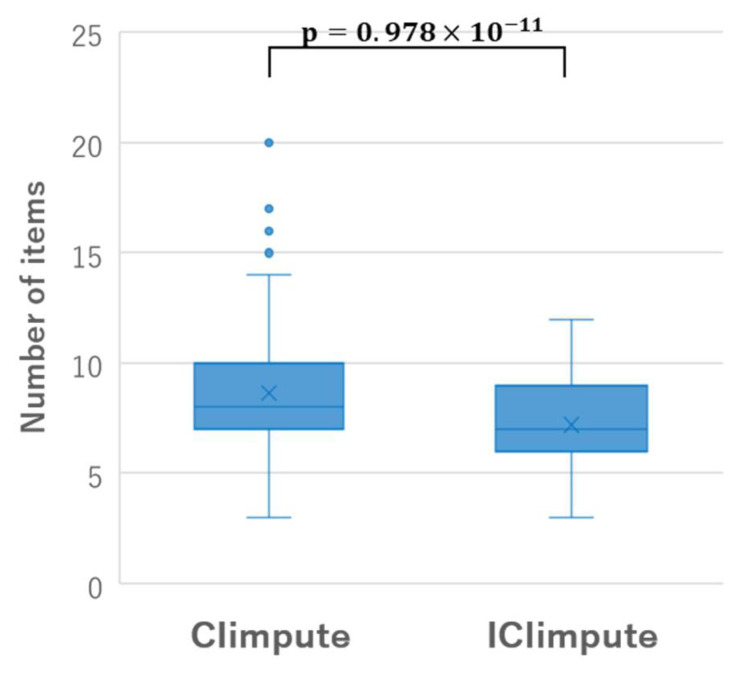
Box plots of the number of items included in the closed itemsets used in the missing value imputation.

**Figure 11:**
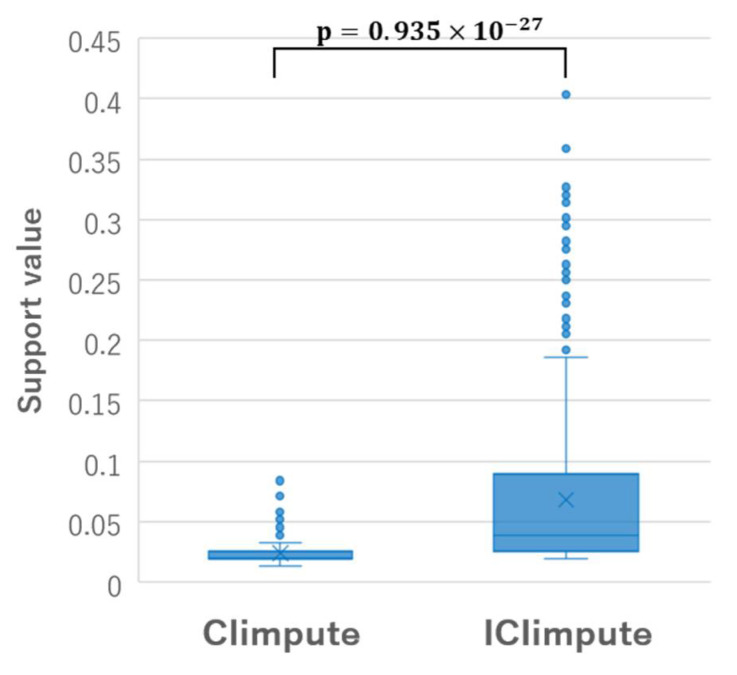
Box plots of the support values of the closed itemsets used in the missing value imputation.

Here, through an application to the Parkinson dataset, we discuss the difference between CIimpute and ICIimpute from the perspective of the characteristics of the closed itemsets used for missing value imputation. Figure 10 shows the box plots of the number of items included in the closed itemsets used for missing value imputation of CIimpute and ICIimpute. As can be seen, the number of items included in the closed itemsets used in ICIimpute tends to be fewer than that of CIimpute. This is because the number of available attributes decreased by the attribute reduction process. Figure 11 shows the box plots of the support values of the closed itemsets used for missing value imputation of CIimpute and ICIimpute. From this figure, we can see that the support values of the closed itemsets used in ICIimpute tend to be significantly larger than those of CIimpute. This means that closed itemsets covering more samples contribute to better missing value imputation. Although we have discussed here the number of items and the support values of the closed itemsets, in future, it will be necessary to investigate other characteristics of the closed itemsets, such as the composition of items and class specificity. We expect that these investigations will contribute to further improvement in imputation accuracy.

**Figure 12:**
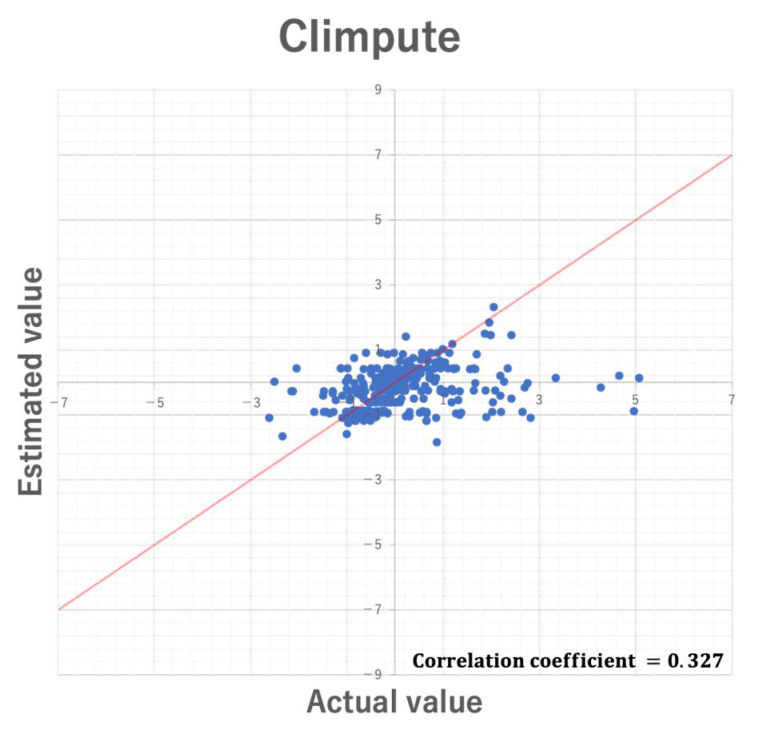
Scatter plot of the estimated values by CIimpute and the actual values.

**Figure 13:**
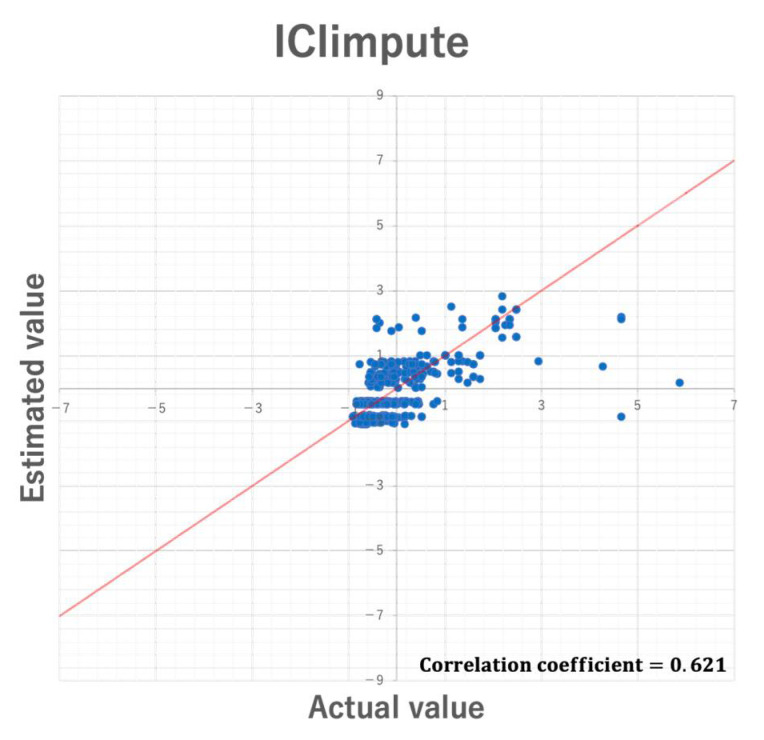
Scatter plot of the estimated values by ICIimpute and the actual values.

In the experiment, RMSE was used to compare the imputation accuracy of the two proposed methods. However, although RMSE allows relative comparison of the imputation accuracy, it does not always guarantee unbiased imputation. Further, such bias may be affected by datasets used for missing value imputation. Here, we discuss the bias of estimated values by 5-fold cross validation using the Parkinson dataset. Figure 12 and Figure 13 show the scatter plots of the actual values and the estimated values in CIimpute and ICIimpute, respectively. As you can see, ICIimpute can provide better estimated values that are closer to the actual values than CIimpute. However, in both methods there exist many estimated values that differ substantially from the actual values. In order to realize more accurate estimation of missing value, it is necessary to improve the calculation method of estimated value, i.e., Equation (4), and investigate the characteristics of closed itemsets that are effective for missing value imputation.

Jin et al. [21] compared the performance of seven state-of-the-art missing value imputation methods using a large-scale benchmark dataset and immune cell dataset. The results showed that the random forest-based method (RF) showed the best imputation accuracy. In the result in Section 5.3.1, ICIimpute demonstrated imputation accuracy higher than or comparable to that of RF. In particular, in the segmentation dataset, the difference in the accuracy between ICIimpute and RF was significantly large. The segmentation dataset consisted of a large number of classes (seven classes) compared to the other datasets (two classes). RF has not supported missing value imputations for multiclass datasets. In contrast, our approach performed missing value imputation using closed itemsets that occurred in each class. Consequently, we consider that the effect of our approach became more prominent in the segmentation dataset for which the number of classes was large. On the other hand, compared to existing methods, the proposed methods incur significant computational costs. This is a serious disadvantage when applying the proposed methods to large-scale real-world data. However, we believe that the search for closed itemsets can be made more efficient by introducing pruning techniques. For example, a previous study [22] implemented a pruning technique for the LCM algorithm, which rapidly searches for closed itemsets that appear only in each class. Such efficient and fast pruning techniques will be an effective way to address the disadvantages of the proposed methods.

Missing value is generally divided into three mechanisms, missing completely at random (MCAR), missing at random (MAR), and missing not at random (MNAR). MCAR is a situation that the probability of an observation being missing is independent of observed or unobserved measurements. MAR is a situation that the probability of an observation being missing depends only on observed measurements. MNAR is a situation that the probability of an observation being missing depends on unobserved measurements. In this study, we assumed the MCAR situation and that estimated missing values were generated completely at random. However, MCAR is the most unrealistic assumption among the three mechanisms. To show realistic availability, in future, we will conduct missing value imputation experiments under MAR and MNAR situations, for example, using the datasets in the literature [21] or [23]. However, we need some important modifications in the proposed method to address MAR and MNAR situations. The proposed methods performed missing value imputation using an only attribute containing the missing value in the closed itemset. In order to address the mechanisms of MAR and MNAR, it will be necessary to use the data distribution of the other attributes in the closed itemset as well as the attribute containing the missing value. In addition, to obtain more accurate and unbiased estimated values, we need to introduce processes for correcting imputed values, such as the bias-corrected estimator proposed in [24].

The advantages of the proposed methods are as follows.

- It is possible to estimate missing values using local feature space for multiclass matrix datasets.
- It is possible to provide more accurate estimated values that are robust to variation of missing rate compared to the existing methods.

The limitations of the proposed methods are as follows.

- It requires more computational time compared to the existing methods.
- It requires further modifications to apply to MAR and MNAR.

## 6. Conclusions

In this paper, we have presented two missing value imputation methods, CIimpute and ICIimpute, based on closed itemsets for multiclass matrix data. The proposed methods enable us to estimate missing values based on data patterns of local feature space in matrix data. CIimpute estimated missing values using closed itemsets extracted from each class. ICIimpute introduced attribute reduction to CIimpute. We applied the proposed methods to four USI datasets and evaluated their imputation accuracy and computational time.

First, we compared CIimpute and ICIimpute, with various reduction rates and missing rates, and found that ICIimpute showed superior performance for both the imputation accuracy and computational time, which indicates that attribute reduction was effective. Second, we compared ICIimpute to three existing methods, KNNimpute, LSimpute, and RF. The results revealed that ICIimpute provided better imputation accuracy; however, it required more computational time. This result suggests that ICIimpute requires further improvement to reduce the computational time.

In future, we will extend the proposed method to apply to MAR and MNAR. In addition, following the literature [22], we will implement a pruning method to closed itemset mining to reduce the computational cost. Furthermore, we will apply the proposed methods to real data, such as image, audio, and gene expression data.

## Figures and Tables

**Table 1:** UCI datasets used in the experiments.

Dataset	# of Attributes	# of Samples	Class Label	# of Samples in Each Class
Parkinson	23	197	1	49
			2	148
SPECTIF	44	80	1	30
			2	50
segmentation	19	210	1	30
			2	30
			3	30
			4	30
			5	30
			6	30
			7	30
acoustic	46	240	1	121
			2	119

**Table 2:** Computational times for ICIimpute and three existing methods.

Methods	Parkinson	SPECTIF	Segmentation	Acoustic
Method2	3628	6537	11,384	16,827
KNNimpute	11	9	11	10
LSimpute	18	14	N/A	22
RF	67	68	64	261

## Data Availability

The program code used in the research can be obtained from the corresponding author upon request.
